# Photosynthesis and Growth of *Pennisetum centrasiaticum* (C_4_) is Superior to *Calamagrostis pseudophragmites* (C_3_) during Drought and Recovery

**DOI:** 10.3390/plants9080991

**Published:** 2020-08-04

**Authors:** Yayong Luo, Xueyong Zhao, Ginger R. H. Allington, Lilong Wang, Wenda Huang, Rui Zhang, Yongqing Luo, Zhuwen Xu

**Affiliations:** 1 Naiman Desertification Research Station, Northwest Institute of Eco-Environment and Resources, Chinese Academy of Sciences, Lanzhou 730000, China; zhaoxy@lzb.ac.cn (X.Z.); wanglilong@lzb.ac.cn (L.W.); huangwd@lzb.ac.cn (W.H.); zhangrui@lzb.ac.cn (R.Z.); luoyongqing@lzb.ac.cn (Y.L.); 2 Laboratory of Stress Ecophysiology and Biotechnology, Northwest Institute of Eco-Environment and Resources, Chinese Academy of Sciences, Lanzhou 730000, China; 3 Department of Geography, The George Washington University, Washington, DC 20052, USA; gallington@email.gwu.edu; 4 Ministry of Education Key Laboratory of Ecology and Resources Use of the Mongolian Plateau, Hohhot 010021, China

**Keywords:** psammophytes, photosynthesis, biomass, drought, recovery

## Abstract

Global warming and changes in rainfall patterns may put many ecosystems at risk of drought. These stressors could be particularly destructive in arid systems where species are already water-limited. Understanding plant responses in terms of photosynthesis and growth to drought and rewatering is essential for predicting ecosystem-level responses to climate change. Different drought responses of C_3_ and C_4_ species could have important ecological implications affecting interspecific competition and distribution of plant communities in the future. For this study, C_4_ plant *Pennisetum centrasiaticum* and C_3_ plant *Calamagrostis pseudophragmites* were subjected to progressive drought and subsequent rewatering in order to better understand their differential responses to regional climate changes. We tracked responses in gas exchange, chlorophyll fluorescence, biomass as well as soil water status in order to investigate the ecophysiological responses of these two plant functional types. Similar patterns of photosynthetic regulations were observed during drought and rewatering for both psammophytes. They experienced stomatal restriction and nonstomatal restriction successively during drought. Photosynthetic performance recovered to the levels in well-watered plants after rewatering for 6–8 days. The C_4_ plant, *P. centrasiaticum*, exhibited the classic CO_2_-concentrating mechanism and more efficient thermal dissipation in the leaves, which confers more efficient CO_2_ assimilation and water use efficiency, alleviating drought stress, maintaining their photosynthetic advantage until water deficits became severe and quicker recovery after rewatering. In addition, *P. centrasiaticum* can allocate a greater proportion of root biomass in case of adequate water supply and a greater proportion of above-ground biomass in case of drought stress. This physiological adaptability and morphological adjustment underline the capacity of C_4_ plant *P. centrasiaticum* to withstand drought more efficiently and recover upon rewatering more quickly than *C. pseudophragmites* and dominate in the Horqin Sandy Land.

## 1. Introduction

Global warming and changes in rainfall patterns may put many ecosystems at risk of drought or even extreme drought, and many arid regions of the globe projected to be dryer and more variable under climate change [1]. On the other hand, psammophytes (plants that like/adapt to live in sand-based areas), dominate in semiarid sandy land areas where scarce precipitation occurs and water is easily lost through evaporation and percolation, which have evolved certain mechanisms, such as effective defensive systems and “fast growing” strategy, to enable them to survive and assimilate as much as possible for coping with frequent soil droughts [2]. Therefore, understanding responses of plant photosynthesis and growth to drought and rewatering after the drought is essential, especially in arid and semiarid ecosystems [3,4].

Photosynthesis powers life on earth using sunlight to fix carbon dioxide. The photosynthetic response of C_3_ plants to drought stress have been well studied and reviewed [5,6,7], whereas the response of C_4_ photosynthesis to water stress has not received due attention [8,9]. C_4_ plants are subject to all major parameters of global change, often in a different manner to C_3_ plants [10]. Although C_4_ plants make up only 4% of the world’s flora, they contribute about 20% of global primary productivity [8]. In addition, C_4_ plants grow mainly in hot, arid areas where droughts are frequent, so it is important to understand the photosynthesis characteristics of C_4_ plants responds to drought [8,10], in particular, what is the photosynthetic and growth response?

Responses of trees and crops to drought and rewatering are well studied [11,12]; vegetative growth of drought-stressed plants can recover after drought [7,13,14], suggesting a reversibility of physiological changes induced by water deficiency. However, specific strategies are less well known about the psammophytes responding to drought and rewatering in sandy lands, especially for the comparison of C_3_ and C_4_ plant. Moreover, C_3_ and C_4_ psammophytes coexist in the semiarid sandy land, which is prone to frequent drought in the growing season. However, the answer to whether C_4_ plants get prosperous at the expense of C_3_ species is not clear, especially in the case of frequent extreme drought events.

The Horqin Sandy Land is one of the most serious areas of desertification in China, while the desertification is reversed as a whole in this region, with the implementation of restoration measures [15,16]. C_4_ plant (*Pennisetum centrasiaticum*) and C_3_ plant (*Calamagrostis pseudophragmites*) are two dominant psammophytic perennial grasses mainly in fixed sandy land, and they are potential dominant species in the later stage of sandy vegetation restoration in Horqin Sandy Land. Previous work has shown that *P. centrasiaticum* is able to withstand extreme drought and recover quickly after rehydration [17]. More recent work has suggested that *P. centrasiaticum* (C_4_) may be able to withstand extreme drought events more efficiently and recover from drought more quickly, compared to *C. pseudophragmites* (C_3_) (results are shown in this paper). Thus, in this work, we perform a direct comparison of the photosynthetic performance and biomass between these two species, representing two functional types, during drought and recovery period. One C_4_ grass (*P. centrasiaticum*) and one C_3_ grass (*C. pseudophragmite*) that co-occur in sandy grasslands of North China were planted withholding water firstly, then rewatering to recovery.

Therefore, the main focus of this study thus is photosynthetic adaptation to drought stress and the recovery from drought. The aim of this paper is to understand the potential ecosystem responses of arid sandy grasslands under climate change. We investigate this by assessing the differentiation of photosynthetic performance and biomass allocation to drought and rewatering, between C_4_ plant *P. centrasiaticum* and C_3_ plant *C. pseudophragmites*.

## 2. Results

### 2.1. Climatic Conditions and Water Status

The environmental conditions through the experiment were typical of summer in Horqin Sandy Land. Air temperature photosynthetic active radiation (PAR), relative air humidity and photosynthetic active radiation (PAR) on the sampling days ranged between 29.2 and 33.6 °C, 24.4 and 47.6%, and 1085 and 1689 μmol m^−2^ s^−1^, respectively. Meanwhile, ambient CO_2_ concentration ranged between 360 and 390 μmol mol^−1^.

**Figure 1:**
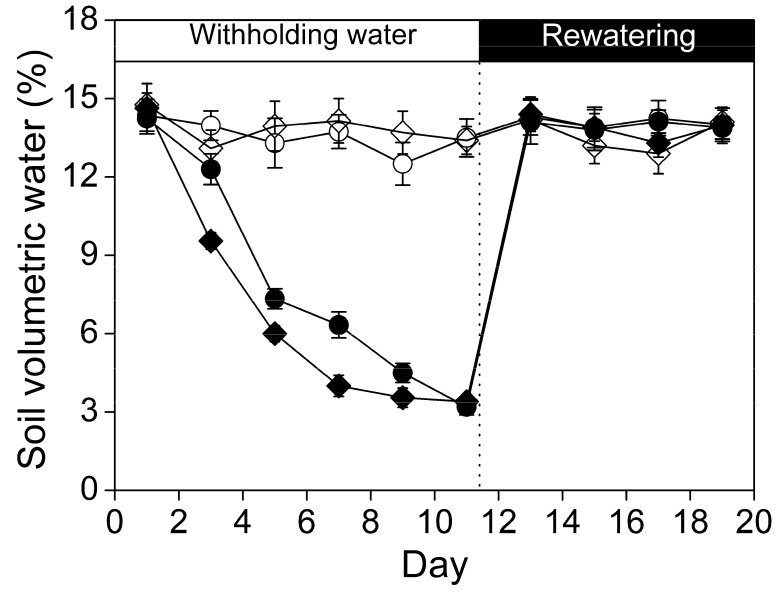
Changes in soil moisture during drought and rewatering for *P. centrasiaticum* (circle) and *C. pseudophragmites* (diamond), respectively. Open circles (○) and diamond (◊) denote the well-watered treatment, and the filled them denote drought-stressed. The rewatering is indicated by the dotted line. Values are means ± SE (*n* = 9).

Soil volumetric water decreased to 3.2% and to 3.4% after withholding water for 11 days in drought-stressed *P. centrasiaticum* and *C. pseudophragmites*, respectively (Figure 1). Soil water loss occurred more slowly in *P. centrasiaticum* than *in C. pseudophragmites*. Upon rewatering, soil moisture was recovered immediately to the well-watered level, which kept stable during rewatering period. Soil moisture fluctuated slightly between 12.5% and 14.4% in *P. centrasiaticum*, and between 12.9% and 14.8% in *C. pseudophragmites* for well-watered throughout the whole experiment (Figure 1).

### 2.2. Gas Exchange Characteristics

**Figure 2:**
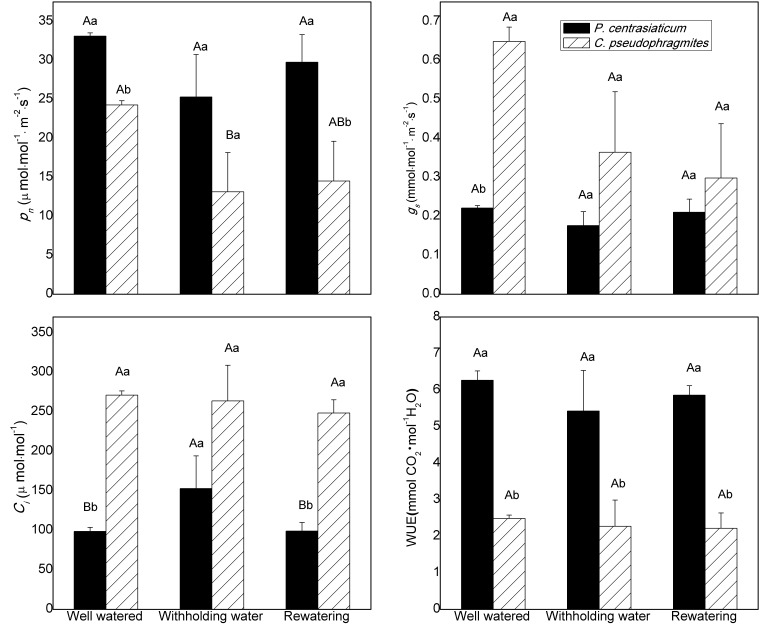
Changes in leaf net photosynthetic rate (*P_n_*), stomatal conductance (*g_s_*), intercellular CO_2_ concentration (*C_i_*) and water use efficiency (WUE) during soil drought and rewatering for *P. centrasiaticum* (C_4_) and *C. pseudophragmites* (C_3_), respectively. Drought-stressed treatment is divided into two periods, namely, withholding water period and rewatering period. There were ten, six and four days of measurement for well-watered, withholding water and rewatering, respectively. Differences among well-watered, withholding water period, and rewatering period were marked with capital letters for each species. Differences between the two species were marked with lowercase letters. Values are means ± SE.

The average values of net photosynthetic rate (*P*_n_) and water use efficiency (WUE) were higher in *P. centrasiaticum* than in *C. pseudophragmites* for across all treatments (*p* < 0.05), with the exception of *P*_n_, which was not different in the drought treatment. However, leaf *g*_s_ and *C_i_* in *P. centrasiaticum* were often lower than in *C. pseudophragmites*, especially in well-watered treatment (*p* < 0.05) (Figure 2). Leaf *P*_n_, *g*_s_ and WUE during drought and rewatering periods were lower than those under well-watered treatments for both species. Leaf *P*_n_ was only suppressed significantly (*p* < 0.05) in *C. pseudophragmites* during the drought treatment period, while there were no significant differences in leaf *g*_s_ and WUE among well-watered, withholding water and rewatering for each species (Figure 2). Meanwhile, leaf *C_i_* increased significantly (*p* < 0.05) in *P. centrasiaticum* during withholding water period, but there was no difference between well-watered and rewatering period in *P. centrasiaticum*, or across any of the treatments in *C. pseudophragmites* (Figure 2).

### 2.3. Chlorophyll Fluorescence Characteristics

**Figure 3:**
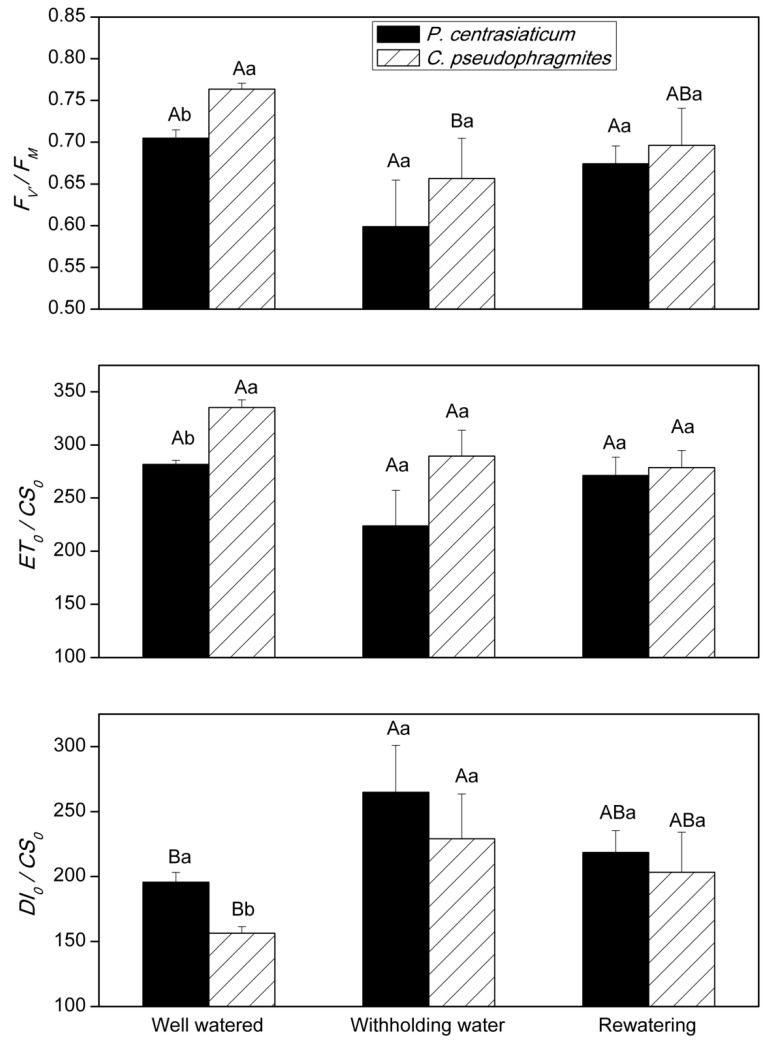
Changes in leaf *F_V_*/*F_M_* (maximum quantum yield of PS II), *ET*_0_/*CS*_0_ (electron transport flux) and *DI*_0_/*CS*_0_ (dissipated energy flux) during soil drought and rewatering for *P. centrasiaticum* (C_4_) and *C. pseudophragmites* (C_3_), respectively. There were ten, six and four days of measurement for well-watered, withholding water and rewatering, respectively. Differences among well-watered, withholding water and rewatering were marked with capital letters. Differences between two species were marked with lowercase letters. Values are means ± SE.

Leaf *F_V_*/*F_M_* and *ET_0_/CS_0_* in well-watered plants and drought-stressed plants were lower in *P. centrasiaticum* than in *C. pseudophragmites* when soil moisture is similar, respectively, although they only had significant differences for well-watered conditions (*p* < 0.01). While leaf *DI_0_/CS_0_* (*p* < 0.05) was higher in *P. centrasiaticum* than in *C. pseudophragmites*, especially in a well-watered treatment (*p* < 0.05) (Figure 3). During drought and the rewatering period, leaf *F_V_*/*F_M_* and *ET_0_/CS_0_* tended to decrease, while leaf *DI_0_/CS_0_* tended to increase for both species. Drought suppressed the *F_V_*/*F_M_* (*p* < 0.05) in *C. pseudophragmites*, while it had no significant suppression in *P. centrasiaticum*. Leaf *DI_0_/CS_0_* was raised significantly (*p* < 0.05) for these two species during drought (Figure 3). However, there were no significant differences in *F_V_*/*F_M_ ET_0_/CS_0_* and *DI_0_/CS_0_* between rewatering and well-watered conditions, and between rewatering and withholding water (Figure 3).

### 2.4. Biomass Characteristics

Both below-ground biomass and total biomass were higher in *P. centrasiaticum* than in *C. pseudophragmites* for well-watered (*p* < 0.01, *p* < 0.05) and drought-stressed conditions (*p* < 0.05, *p* < 0.05), respectively. Above-ground biomass was higher in *P. centrasiaticum* than in *C. pseudophragmites* for drought-stressed (*p* < 0.05) conditions, but it had no significant differences between two species for well-watered conditions (Table 1). Root: shoot ratio was higher for well-watered conditions (*p* < 0.05), while it was lower for drought-stressed conditions (*p* < 0.05) in *P. centrasiaticum*, compared to *C. pseudophragmites*.

Drought stress decreased above-ground, below-ground, and total biomass by a factor 11%, 53% (*p* < 0.01) and 37% (*p* < 0.05) in *P. centrasiaticum*, as well as 73% (*p* < 0.01), 58% (*p* < 0.05) and 66% (*p* < 0.01) in *C. pseudophragmites*, although the difference is not significant for above-ground biomass in *p. centrasiaticum*. Under drought-stressed conditions, root: shoot ratios were reduced by a factor 45% (*p* < 0.05) in *P. centrasiaticum* and increased by 56% (*p* < 0.05) in *C. pseudophragmites* (Table 1).

## 3. Discussion 

### 3.1. Photosynthetic Adjustment and Biomass Response to Drought and Recovery for Two Psammophytes

**Figure 4:**
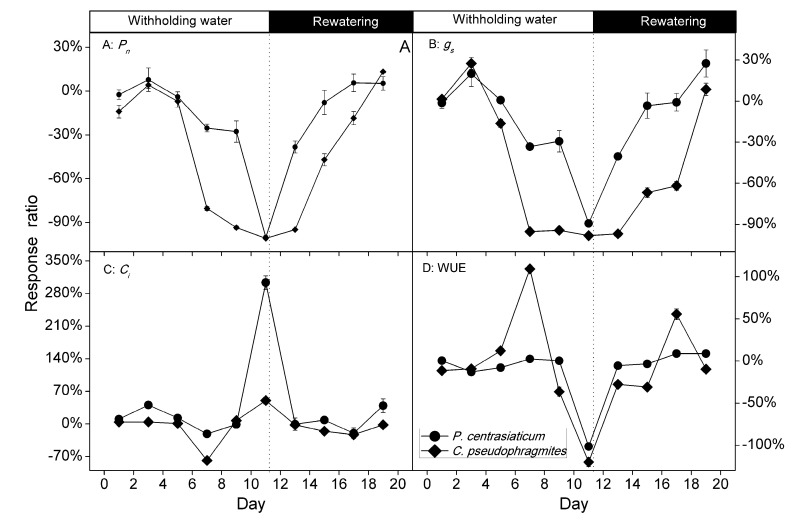
Changes of responses ratio in leaf net photosynthetic rate (*P_n_*), stomatal conductance (*g_s_*), intercellular CO_2_ concentration (*C_i_*) and water use efficiency (WUE) during soil drought and rewatering for *P. centrasiaticum* (C_4_) and *C. pseudophragmites* (C_3_), respectively. Filled circles (●) and diamond (♦) denote *P. centrasiaticum* and *C. pseudophragmites* respectively. The beginning of rewatering is indicated by the dotted line.

**Figure 5:**
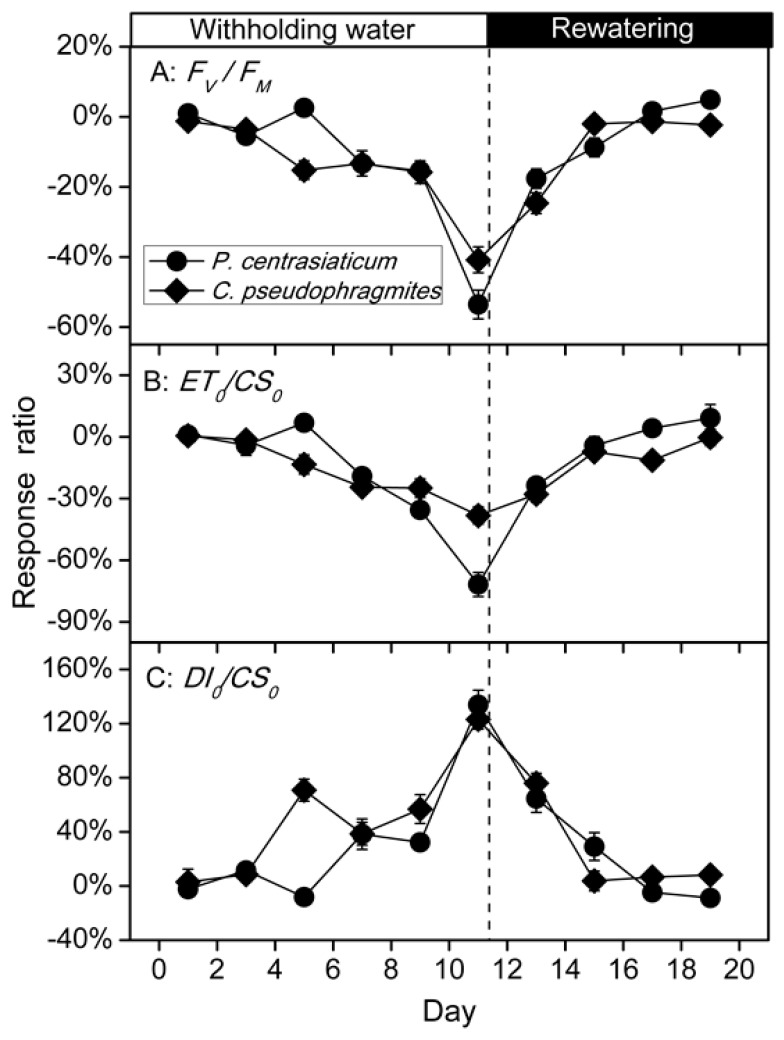
Changes of response ratio in leaf *F_V_*/*F_M_* (maximum quantum yield of PS II), *ET*_0_/*CS*_0_ (electron transport flux) and *DI*_0_/*CS*_0_ (dissipated energy flux) during drought and rewatering period for *P. centrasiaticum* (C_4_) and *C. pseudophragmites* (C_3_), respectively. Filled circles (●) and diamond (♦) denote *P. centrasiaticum* and *C. pseudophragmites* respectively. The beginning of rewatering is indicated by the dotted line.

The response ratio is often used to measure the effect of an experiment because it quantifies the proportional changes generated by the experimental operation [18]. A progressive water limitation was caused by withholding water for 11 days for the two psammophytes (Figure 1, Figure 4 and Figure 5). The severity of drought affects the relative contributions of diffusion and metabolic restrictions, according to leaf gas exchange measurements [5,6]. During the withholding water period, leaf *P*_n_ and *g*_s_ declined, suggesting that stomatal limitation seemed to explain the suppression of photosynthesis. Stomatal closure prevents further water loss and irreversible cell dehydration during soil drought [7]. Meanwhile, *C_i_* increased compared to well-watered treatment. The increased *C_i_* may be the result of patchy stomatal closure and cuticular conductance under drought, and the CO_2_ concentration in chloroplasts of stressed leaves was lower than *C_i_* caused by decreasing mesophyll conductance, especially at very low *g*_s_ [5]. These results indicated that predominance of nonstomatal limitations to photosynthesis during drought periods for these species in the late drought [19].

Chlorophyll fluorescence is often used to detect the flow of excitation energy within PS II and provide insight into the mechanisms of photosynthetic regulation [20,21]. It can be used to get information about the efficiency of electron transmission through PS II [22,23]. *F_V_*/*F_M_* and *ET_0_/CS_0_* reduced, while *DI_0_/CS_0_* increased under the withholding water and rewatering periods (Figure 3). This result indicated that the trapping probability and the electron transport capacity of PS II was suppressed [21], which also indicated that the decrease of photosynthesis in late drought was mainly related to nonstomatal limitation [24]. Additionally, heat dissipation increased during both drought and rewatering stages when *P*_n_ was suppressed (Figure 2 and Figure 3). These results suggested a flexible regulation for capture and transfer of excitation energy within PS II in psammophytes, resulting in an more electron flow to substitute sinks, which is consistent with previous studies [7,20]. Both reversible downregulation of PS II photochemistry and enhancement of heat dissipation excess excitation energy (*DI_0_/CS_0_*) contributed to enhanced photo-protection in drought-stressed plants [7].

Studies have shown that degree and duration of drought influence the recovery rate following stress relief [12,25]. The recovery pattern of drought-stressed *P. centrasiaticum* and *C. pseudophragmites* was similar (Figure 4 and Figure 5). Recovery of gas exchange and chlorophyll fluorescence characteristics was observed following two to six days of rewatering, which was consistent with previous studies [7,12]. The delayed recovery of photosynthetic performance of drought-stressed plants, these two psammophytes during rewatering periods may be an adaptive strategy in Horqin Sandy Land where drought occurred frequently. The strategy contributes to minimize water loss during rewatering and reduce future drought stress [7].

The recovery fraction of gas exchange and chlorophyll fluorescence characteristics were similar, but the speed was different for both species (Figure 4 and Figure 5). For example, the recovery speed of *C_i_* was faster than that of *P_n_* and *g_s_*, suggesting that metabolic restrictions inhibited photosynthesis more than diffusive limitation [5,7] in the initial stage of hydration (data not shown).

In addition, drought-induced changes on leaf morphology or biomass may also play an important role in adaptation to drought [26]. Drought and rewatering reduced total and below-ground biomass compared to a well-watered treatment, for two species (Table 1).

### 3.2. Differentiation of Photosynthetic Performance and Biomass to Drought and Rewatering between Two Psammophytes

Averaged leaf *P*_n_ in C_4_ plant *P. centrasiaticum* was higher than that in C_3_ plant *C. pseudophragmites*, while leaf *g*_s_ and *C_i_* in *P. centrasiaticum* were lower than those in *C. pseudophragmites* for well-watered, withholding water and rewatering treatments, respectively (Figure 2). The results revealed that *P. centrasiaticum* kept lower stomatal opening and more efficient CO_2_ assimilation even under sufficient moisture conditions compared to *C. pseudophragmites*, which is beneficial to reduce moisture loss and increase leaf *P*_n_ (Figure 2) [8]. As a result, Leaf WUE in *P. centrasiaticum* were more than twice as high as in *C. pseudophragmites* (Figure 2). This observation agrees with the general theory that the CO_2_-concentrating mechanism in the leaves of C_4_ plants helps them by having higher photosynthetic rate and water use efficiency than C_3_ plants [7,9,10].

Compared to the well-watered treatment, the average leaf *P*_n_, *g*_s_, WUE, *F_V_*/*F_M_* and *ET_0_/CS_0_* in C_4_ plant *P. centrasiaticum* decreased by 25%, 22%, 20%, 14% and 20%, respectively; which in C_3_ plant *C. pseudophragmites* decreased by 49%, 46%, 9%, 15% and 17%, respectively, through the 11 days of drought period. That is, response ratios of *P*_n_ and *g*_s_ in *P. centrasiaticum* (C_4_) were lower than in *C. pseudophragmites* (C_3_) under the similar drought intensities, which further validates the conclusion using meta-analysis [3]. The capacity of stoma regulation in *C. pseudophragmites* was higher than that in *P. centrasiaticum* (C_4_), which resulted in higher fluctuation of WUE (Figure 4). Additionally, average leaf *C_i_* increased by 58% in *P. centrasiaticum* (C_4_), but it decreased by 2% in *C. pseudophragmites* (C_3_) under the withholding water period. Therefore, the dependence of stoma regulation in *P. centrasiaticum* (C_4_) was lower than *C. pseudophragmites* (C_3_). Meanwhile, average leaf *DI_0_/CS_0_* increased by 34% and 50% in *P. centrasiaticum* and *C. pseudophragmites* from well-watered to withholding water, respectively (Figure 3), which indicated the *C. pseudophragmites* (C_3_) had a more pronounced response to drought stress regarding the thermal dissipation than *P. centrasiaticum* (C_4_). However, the larger relative increase of thermal dissipation did not remove excess excitation energy enough, and it did not mitigate the rapid decline in *P*_n_ under the drought period, compared to *P. centrasiaticum* (C_4_) (Figure 2 and Figure 4). Additionally, the absolute value of energy removed by thermal dissipation was lower (not significant for drought and rewatering treatment) in *C. pseudophragmites* (C_3_) than in *P. centrasiaticum* (C_4_) (Figure 3). As a result, less excitation energy was transferred into photosynthetic apparatus help to alleviate drought stress in *P. centrasiaticum* (Figure 2 and Figure 3). In general, *P*_n_, *g*_s_ and *ET_0_/CS_0_* in C_4_ plant *P. centrasiaticum* decreased less and maintained their photosynthetic advantage until water deficits became severe and responded with greater metabolic limitations compared to C_3_ grass *C. pseudophragmite* in the withholding water period (Figure 4 and Figure 5), which is consistent with the previous reports [24,25].

During rewatering for 8 days, average leaf *P*_n_, *g*_s_, WUE, *F_V_*/*F_M_* and *ET_0_/CS_0_* in *P. centrasiaticum* decreased by 9%, 4%, −2%, 5% and 4% 21%, respectively, which in *C. pseudophragmites* decreased by 37%, 54%, 3%, 8% and 12%, respectively, compared to well-watered treatment (Figure 2, Figure 3, Figure 4 and Figure 5). These results indicated that photosynthetic performance was recovered more quickly in *P. centrasiaticum* than that in *C. pseudophragmites*. However, Ripley et al. reported that C_4_ plants recovered more slowly on rewatering due to their drought-sensitive metabolism, compared to C_3_ plants [27]. Earlier studies showed that the recovery speed of photosynthetic parameters was different among species and those parameters [7,13], thus the differences may be specific in species or stress and require more study.

Due to higher leaf *P*_n_ and quicker recovery after rewatering, the above-ground biomass, below-ground biomass and total biomass were higher in *P. centrasiaticum* than those in *C. pseudophragmites* for both water treatments, respectively (Table 1). In general, plants usually allocate a larger proportion of biomass to their roots to absorb more moisture under drought-stressed conditions [28], resulting in an increase in root–shoot ratio. However, root: shoot ratios were reduced in *P. centrasiaticum* but increased in *C. pseudophragmites* under drought and rewatering conditions (Table 1 and Table 2). Sack et al. reported that leaf hydraulic conductance was disproportionately high, independently of *g*_s_ for C_4_ grasses [29], compared to C_3_ grasses, this “hyper-efficient” water transport as important an adaptation as C_4_ biochemistry, enabling the photosynthetic advantage of them in moist soil and moderate drought. In addition, *P. centrasiaticum* take precautions to distribute more root biomass when there is no shortage of water (Table 1). Therefore, *P. centrasiaticum* can use less root biomass distribution to meet the water needs of plants upon rewatering, which in turn facilitates the recovery of leaves and photosynthetic organs, allowing it to perform competitively in Horqin Sandy Land where precipitation is scarce and soil water is easily lost through evaporation and seepage [27].

In summary, leaf *P*_n_, *g*_s_, WUE, *F_V_*/*F_M_* and *ET_0_/CS_0_* decreased, while *DI_0_/CS_0_* increased under the withholding water and rewatering periods in both plants (Figure 2 and Figure 3, Table 2). Similar patterns of photosynthetic regulation were observed during drought and rewatering for these the two psammophytes. Both of them experienced stomatal restriction and nonstomatal restriction successively during drought. Stomatal closure prevents further water loss and nonreversible cell dehydration. Downregulation of PS II photochemistry and electron transport, as well as an increase of thermal dissipation contributed to the removal of excess excitation energy and photo-protection in drought-stressed plants. As a result, their photosynthetic performance recovered to the levels in well-watered plants after rewatering for 6-8 days. Nevertheless, C_4_ plant *P. centrasiaticum* kept a lower *g*_s_, *C_i_*, *F_V_*/*F_M_* and *ET_0_/CS_0_* and presented higher *P*_n_, biomass, WUE, root: shoot ratio and *DI_0_/CS_0_*, than C_3_ plant *C. pseudophragmites* (Figure 2 and Figure 3, Table 1). The CO_2_-concentrating mechanism in the leaves of C_4_ plant allows for more efficient CO_2_ assimilation and water use efficiency. More efficient thermal dissipation facilitates their ability to alleviate drought stress.

If C_4_ plants rely on high photosynthesis and water use efficiency, they can allocate a greater proportion of root biomass in case of adequate water supply and a greater proportion of above-ground biomass in case of drought and rewatering. We speculate that C_4_ plants might increase their dominance in the semiarid grassland ecosystem mixed with C_3_/C_4_ plants, in the future climate environment with frequent droughts, but more research is required on more C_4_ plants in this region.

## 4. Materials and Methods

This study was carried out in the southwest of Horqin Sandy Land (42°55′ N, 120°44′ E; about 360 m ASL), located in the northeast of Inner Mongolia, Northern China. The climate in this region is temperate, semiarid and continental monsoonal. The mean annual precipitation is 343 mm, with 75 percent of it occurring between June and September. The average annual latent evaporation is 1935 mm. Psammophytes are the native flora, such as *Agriophyllum squarrosum, Setaria viridis, P. centrasiaticum*, *C. pseudophragmites, and Artemisia halodendron*.

Budding *P. centrasiaticum* root segments and underground budding *C. pseudophragmites* from the sand dune community were transplanted into sandy plastic pots with 27.6 cm diameter and 26.5 cm depth on April 22, 2009. Before transplanting, 20 g of slow-release fertilizer with NPK (14:14:14) was added to each pot, which corresponds to the properly restored sand’s soil nitrogen level. For each species, 30 potted plants with holes in the bottom were placed in the open air and irrigated until water treatments began when the leaves were completely developed. In a completely random design, 18 pots with uniform plants were treated in two different treatments for each species, both well-watered and drought-stressed [7]. All plants were arranged under a mobile awning, which covered the plants when it rained, and then were moved away after rain. For the duration of the experiment, well-watered plants were watered daily to the field capacity. Stressed plants were withheld from water for 11 days until the net photosynthetic rate (*P_n_*) approached 0 during late morning then watered daily until the *P_n_* recovered [7,17]. For each treatment, six potted plants were used for measuring gas exchange and chlorophyll fluorescence every two days for 20 days (13 June to 2 July), and randomly selected five from six pots were used to measure them; the remaining three potted plants were harvested at the end of the experiment on July 6 to determine their biomass.

### 4.1. Soil Moisture and Climatic Conditions

Soil moisture at 0–20 cm depth was determined in the early morning using TRIME-PICO TDR (Imko Company, Ettlingen, Germany) in nine pots for each treatment per species. Climatic conditions, such as photosynthetic active radiation (PAR), were recorded using a LI-6400 photosynthesis system (LI-COR Biosciences, Lincoln, Nebraska, USA).

### 4.2. Leaf Gas Exchange Measurement

The *P_n_*, transpiration rate (*T_r_*), stomatal conductance (*g_s_*) and intercellular CO_2_ concentration (*C_i_*) were determined from 9:00 to 11:00 using LI-6400. The measurements were conducted on the most recently fully-expanded and unshaded leaves of five plants, with each treatment under control conditions of 30 °C as a block temperature and 1500 μmol m^−2^ s^−1^ f PAR. Their strip-shaped leaves are measured in rectangles in the middle part, so the leaf area measured by LI-6400 can be determined by measuring the width of the middle part of the leaf. Water use efficiency (WUE) of leaves were determined by calculating ratio of *P_n_* to *T_r_*.

### 4.3. Chlorophyll Fluorescence Measurement

On the same leaves that the gas exchange was determined, the chlorophyll fluorescence was measured by Handy-PEA (Hansatech, Norfolk, UK). Dark adaptation was performed for 20 min, followed by measurements for one second under excitation light of 3000 μmol m^−2^ s^−1^.

### 4.4. Analysis of the Chlorophyll Fluorescence Transients: JIP-test

Using the fluorescence transient data, the JIP-test can quantify the progressive flow of energy through Photosystem II (PS II). Partial JIP-test parameters (Table 3) were showed mainly to explain the distribution of the absorbed energy flux, which contain trapping and dissipation. The F_V_/F_M_ ratio, *ET*_0_/*CS*_0__,_ and *DI*_0_/*CS*_0_ represent trapping probability (TR_0_/ABS), electron transport flux, and dissipation flux, respectively [20,21]. Cross-section parameters are calculated to determine the distribution of energy flux for electron transport and dissipation.

### 4.5. Biomass Measurement

Three pots were selected for each treatment, and both above- and belowground biomass were determined after oven-drying to constant mass at 80 °C.

### 4.6. Statistic Analysis

Changes of gas exchange and JIP-test parameters were grouped according to water treatment and processing period for each species. That is well-watered treatment, withholding water and rewatering period in a drought-stressed treatment. There were ten, six and four days of measurement for well-watered, withholding water and rewatering, respectively. Photosynthesis parameter data were compared with analysis of variance among the watering treatments. Treatments were compared with Student’s t-test analyses between two plants for each period.

The response ratio (*R*) provides an appealing index of effect size for many ecological experiments. Experimental drought and rewatering effects were quantified with the response ratio, which was calculated to explain how drought and rewatering experiment (E) affect photosynthesis parameters relative to the mean value for well-watered (C) at the same time, according to: R = (E-C)/C*100% [18].

## 5. Conclusions

The C_4_ plant *P. centrasiaticum* maintains its photosynthetic advantage until water deficits became severe and quicker recovery after rewatering, although the dependence of stoma regulation in *P. centrasiaticum* (C_4_) was lower than *C. pseudophragmites*. Besides, *P. centrasiaticum* takes precautions to distribute more root biomass when there is no shortage of water, and it can use less root biomass distribution to meet the water needs of plants upon rewatering, which in turn, facilitates the recovery of leaves and photosynthetic organs, leading to allocate more proportion of their biomass to the aboveground parts during drought and rewatering period. This physiological adaptability and morphological adjustment underline the capacity of C_4_ plant *P. centrasiaticum* to withstand drought more efficiently and recover upon rewatering more quickly than *C. pseudophragmites* and dominate in the Horqin Sandy Land. The results might be the first part of a future study including the fieldwork that could give us a more complete picture about the role of both of the species representing the C_4_ plant (*P. centrasiaticum*) and C_3_ plant (*C. pseudophragmites*) in the ecosystem’s response to changes in precipitation patterns.

## Figures and Tables

**Table 1:** Biomass and root: shoot ratio for *P. centrasiaticum* and *C. pseudophragmites.* Values are means ± SE (*n* = 3).

Species and Treatment	Above-Ground Biomass (g)	Below-Ground Biomass (g)	Total Biomass (g)	Root: Shoot Ratio
*P. centrasiaticum*, well-watered	21.86 ± 3.07 Aa	35.72 ± 2.54 Aa	57.58 ± 5.11 Aa	1.69 ± 0.21 Aa
*P. centrasiaticum*, drought-stressed	19.45 ± 3.28 Aa	16.74 ± 1.24 Ba	36.19 ± 2.08 Ba	0.93 ± 0.19 Bb
*C. pseudophragmites*, well-watered	18.73 ± 1.09 Aa	17.05 ± 2.87 Ab	35.78 ± 2.99 Ab	0.92 ± 0.17 Bb
*C. pseudophragmites*, drought-stressed	5.07 ± 0.48 Bb	7.12 ± 0.47 Bb	12.19 ± 0.60 Bb	1.44 ± 0.20 Aa

Note: Significant differences between well-watered and drought-stressed were marked with capital letters for *P. centrasiaticum* and *C. pseudophragmites*, respectively. Significant differences between two species were marked with lowercase letters.

**Table 2:** Similarity and differentiation of net photosynthetic rate (*P_n_*), stomatal conductance (*g_s_*), intercellular CO_2_ concentration (*C_i_*), water use efficiency (WUE), *F_V_*/*F_M_* (maximum quantum yield of PS II), *ET*_0_/*CS*_0_ (Electron transport flux), *DI*_0_/*CS*_0_ (Dissipated energy flux) and biomass traits to drought and rewatering, between C_4_ plant *P. centrasiaticum* and C_3_ plant *C. pseudophragmites*.

Species and Treatment	*P*_n_, Biomass (Above-Ground, Below-ground and Total)	WUE	Root: Shoot Ratio	*DI* *_0_/CS_0_*	*g*_s_, *F_V_*/*F_M_* and *ET_0_/CS_0_*	*C_i_*
*P. centrasiaticum*, well-watered	high	high	high	high	low	low
*P. centrasiaticum*, drought-stressed	reduce less	reduce	reduce	increase	reduce	increase more
*C. pseudophragmites*, well-watered	low	low	low	low	high	high
*C. pseudophragmites*, drought-stressed	reduce more	reduce	increase	increase	reduce	reduce less

**Table 3:** JIP-test (Analysis of the Chlorophyll Fluorescence Transients) parameters with interpretations and formulae calculated using fluorescence transient data.

Terms and Formulae	Explanations
*F_V_*/*F_M_* = (*TR*_0_/*RC*)/(*ABS*/*RC*) = *TR*_0_/*ABS* = [1–(*F*_0_/*F_M_*)]	Maximum quantum yield for PS II
*ET*_0_/*CS*_0_= (*ET*_0_/*RC*)/(*ABS*/*RC*)·(*ABS*/*CS*_0_) = (*ET*_0_/*ABS*)·(*ABS*/*CS*_0_)	Electron transport flux per CS
*DI*_0_/*CS*_0_= (*ABS*/*CS*_0_)–(*TR*_0_/*CS*_0_)	Dissipated energy flux per CS

Notes: *ABS*–absorbance; *CS*–cross-section; *TR*–trapping; *DI*–dissipation; *ET*–electron transport; *F*_0_–minimal fluorescence; *F_M–_*maximum fluorescence; *F_V_*/*F_M_*–maximum quantum yield of PS II; PS II–Photosystem II; *RC*–reaction center.
